# An Advanced CNN-Based Framework for the Automated Detection of Uncovered PET in Recycling Streams

**DOI:** 10.3390/polym17131736

**Published:** 2025-06-22

**Authors:** Adnan Miski, Omer Bafail

**Affiliations:** 1 Department of Industrial Engineering, Faculty of Engineering—Rabigh, King Abdulaziz University, Jeddah 21589, Saudi Arabia; 2 Department of Industrial Engineering, Faculty of Engineering, King Abdulaziz University, Jeddah 21589, Saudi Arabia; oabafail@kau.edu.sa

**Keywords:** waste management, plastic contamination, recycling, YOLO, material recovery facilities, sustainability

## Abstract

Contamination in recycling streams represents one of the most pervasive challenges confronting material recovery facilities (MRFs) globally. Among the various contamination sources in recycling streams, liquid contamination from PET bottles presents particularly severe challenges due to its capacity to spread throughout commingled materials. Object detection using neural networks enables detection at the collection stage of single or mixed recycling streams, allowing for targeted application in the early stage of the recycling cycle. YOLO (you only look once) models and other object detection models are beneficial due to their speed and accuracy in detecting multiple objects at once. This study aimed to design a model to detect contaminated PET bottles in real time. Several YOLO variations and model sizes were trained on a custom dataset with 7130 images. YOLOv8l achieved the highest performance, with mAP@0.5:0.95, mAP@0.5, precision, recall, and F1 score values of 89.7%, 93%, 89%, 88%, and 88%, respectively.

## 1. Introduction

Recycling systems worldwide face significant challenges related to contamination [1,2,3,4,5,6,7,8,9,10], which substantially undermines efficiency and compromises the quality of recycled materials [11,12,13].

In recent decades, single-stream recycling (SSR) has emerged as the predominant approach to municipal waste reclamation in the United States, experiencing unprecedented adoption rates owing to its dual advantages: the simplified participation experience it offers residential users and the logistical efficiencies it provides waste management entities [14]. Despite its widespread implementation, SSR faces significant operational challenges that threaten its environmental and economic viability. The global recycling industry, which processes approximately 370 million tons of materials annually, confronts concerning contamination rates in material recovery facilities (MRFs) inbound recycling streams where contamination levels consistently range from 16% to 50% in SSR streams [15,16,17]. Similar contamination challenges plague alternative collection methods such as mixed waste recycling (MWR) and commingled collection systems (MCS), suggesting that the fundamental act of combining diverse recyclable materials inherently compromises stream purity regardless of the specific implementation approach. This contamination dichotomy, increasing participation rates while simultaneously compromising material quality, represents the central paradox that recycling operators must navigate as they balance accessibility with processing effectiveness in contemporary waste management systems.

When recyclable materials are mixed together in collection bins, as occurs in SSR and MWR, the risk of cross-contamination increases dramatically. This mixing creates several serious problems. Liquids from uncovered bottles and containers soak into paper and cardboard, making them unusable for recycling [18]. Broken glass pieces become stuck in other materials, contaminating them [18]. Different materials touching each other can cause chemical reactions that damage recyclable items. Research by Pressley et al. [19] shows that mixing recyclables leads to contamination rates more than one and a half times higher than when materials are kept separate. Paper products are especially vulnerable, with liquid contamination reducing their recyclability by nearly half. The problem worsens during processing, as machines crushing and sorting the materials can cause additional contamination. MRFs face growing challenges trying to clean these mixed materials to meet strict quality standards, particularly since China implemented its National Sword policy, requiring extremely low contamination levels of just 0.5% [10].

Despite technological advancements in recycling infrastructure, the fundamental challenge of removing liquid-containing containers early in the sorting process remains inadequately addressed. Conventional approaches primarily rely on density-based separation methods, which cannot efficiently isolate uncapped bottles before they contaminate paper and cardboard streams. The correlation between contamination rates and the rejection of recycled materials by end markets has become increasingly evident [20]. For instance, G7 nations, which previously exported 60% of their recyclable plastics to China, faced severe market disruption when China’s 2017 import restrictions reduced acceptance rates to merely 10% and accepted a 0.5% contamination rate. This policy shift created immediate challenges for developed economies dependent on China’s processing capacity. Australia, for example, had been exporting 1.25 million tons of recovered materials annually to China (2016–2017), representing 60% of its paper fiber and 20% of its plastics. Similarly, the United States, which had directed 60% of its recyclables to Chinese markets, suddenly needed to find alternative destinations due to the low quality of those recyclables [10].

### 1.1. Problem Statement

Contamination in recycling streams represents one of the most pervasive challenges confronting material recovery facilities (MRFs) globally, significantly undermining the quality and marketability of processed recyclables. When non-recyclable or improperly prepared items enter the recycling stream, they introduce impurities that compromise material integrity throughout the separation process. These contaminants damage sorting equipment, increase operational costs, and reduce the yield of marketable commodities.

Among the various contamination sources in recycling streams, liquid contamination presents particularly severe challenges due to its capacity to spread throughout commingled materials. For instance, the presence of uncovered plastic bottles containing liquids in recycling streams constitutes a significant challenge for recycling facilities worldwide. These containers, when processed alongside paper, cardboard, and other recyclable materials, release their liquid contents, resulting in widespread contamination. This contamination reduces the quality and value of recycled materials, increases processing costs, and may ultimately lead to the diversion of potentially recyclable materials to landfills. Current automated sorting technologies lack the capability to reliably detect and segregate uncovered liquid-containing bottles at the early stages of the recycling process before contamination occurs.

**Figure 1:**
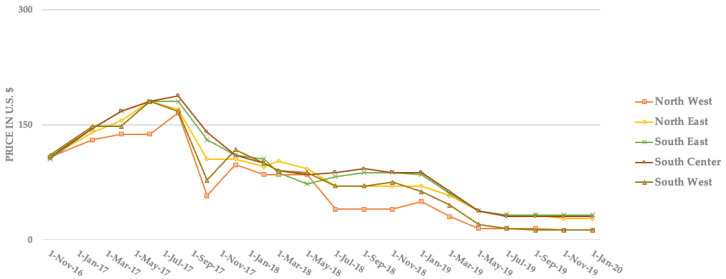
Average prices of recovered OCC bales from 2016 to 2020 in different U.S. regions.

The economic implications of this problem extend beyond the immediate processing challenges. Contaminated materials command lower market prices, with a typical reduction of 40–60% in value compared to uncontaminated materials. For example, most cities in the United States have switched to SSR, which grew from serving just 22% of recycling programs in 2005 to 73% by 2014 [21]. This approach puts all recyclables (paper, plastics, glass, and metals) into one bin, making recycling easier for residents but creating serious contamination problems. When these materials mix, paper becomes wet from leftover liquids in containers, tiny glass pieces stick to other recyclables, and incompatible materials contaminate each other. Figure 1 clearly shows the financial consequences of this contamination problem. After China implemented stricter quality standards in 2017, prices for old corrugated cardboard (OCC) crashed dramatically across all U.S. regions, falling from about USD 150 per ton to just USD 15–37 per ton by 2019, a 75–90% drop in value [10]. This price collapse demonstrates how the convenience of single-stream collection has created contamination levels that threaten both the economic viability of recycling programs and their environmental benefits. This price collapse has substantially increased the risk of these materials ending up in landfills or undergoing incineration. Such outcomes pose a considerable threat to the environment. Landfills, for instance, can contaminate soil and groundwater, potentially impacting nearby agricultural lands through leachate runoff. Incineration releases pollutants into the atmosphere, which can then deposit onto crops and soil, potentially affecting food safety and agricultural productivity. Therefore, the convenience of single-stream collection, by contributing to higher contamination levels, not only threatens the economic viability and environmental benefits of recycling programs but also elevates the likelihood of detrimental impacts on both the broader environment and the agricultural sector.

Additionally, recycling facilities incur higher operational costs due to increased cleaning requirements, equipment maintenance, and waste management expenses associated with rejected materials. The environmental consequences include increased landfill usage and the associated methane emissions, as well as the unnecessary consumption of virgin resources when contaminated recyclables cannot be effectively processed.

### 1.2. Study Objective

The primary objective of this paper is to develop and validate an automated detection system based on convolutional neural networks (CNNs) capable of identifying uncovered polyethylene terephthalate (PET) containing liquids in the collection stage of single or mixed recycling streams. Specifically, this paper aims to achieve the following:

1. Fine-tune and optimize a CNN architecture for detecting uncovered liquid-containing PET in diverse recycling stream environments, which would enable the early identification and removal of these high-risk contaminants before they release their contents onto other recyclables, preventing the cascading contamination that significantly reduces the quality and market value of paper, cardboard, and other recyclables.
2. Train and validate the model using a comprehensive dataset of images representing various bottle types, fill levels, and lighting conditions.
3. Evaluate the model’s performance in terms of detection accuracy, precision, recall, and processing speed under conditions that simulate real-world recycling operations.
4. Develop a practical implementation framework for integrating the detection system into existing recycling facility infrastructure.

### 1.3. Significance of the Study

This paper addresses a critical gap in the current recycling technology by providing a targeted solution to one of the most persistent contamination sources in recycling streams. The significance of this study extends across multiple dimensions:

1. The application of deep learning to address specific contamination issues represents a significant technological leap for the recycling industry, which has traditionally relied on mechanical and optical sorting methods with limited capacity for fine-grained detection.
2. By reducing contamination at the collection stage, this study contributes to higher-quality recycled materials, potentially increasing recycling rates and reducing landfill usage.
3. Each percentage point improvement in contamination rates translates to approximately 3.7 million tons of materials diverted from landfills annually on a global scale.
4. The proposed detection system offers substantial economic advantages for recycling facilities through increased material value, reduced processing costs, and lower rejection rates. Industry analyses suggest that reducing contamination by just 5% could increase the market value of recycled materials by USD 250–300 million annually in North America alone [22].
5. Early detection and removal of PET contaminants streamline processing, reduce equipment wear, and minimize system downtime, thereby enhancing overall operational efficiency.
6. The development of more effective contamination detection technology supports increasingly stringent quality standards in global recycling markets and provides technological solutions to meet emerging regulatory requirements.
7. Reducing contamination in recycling streams directly decreases the volume of materials diverted to landfills, consequently minimizing landfill incineration practices. This reduction in burning waste significantly improves environmental conditions by limiting toxic ash and chemical leachate that would otherwise contaminate agricultural soils and groundwater aquifers. Preserved soil fertility and cleaner groundwater resources enhance agricultural productivity and protect public health in surrounding communities

### 1.4. Motivation of the Study

The motivation for this study stems from several converging factors related to sustainability challenges, technological opportunities, and economic imperatives in the recycling sector. The global recycling landscape has undergone significant transformation in recent years, particularly following policy changes in major recycled material importing countries. China’s National Sword policy implemented in 2018, which banned the import of most plastic waste and set a strict 0.5% contamination limit for acceptable materials, fundamentally disrupted global recycling markets [23,24,25].

This policy shift highlighted the critical importance of contamination reduction in recycling streams and created an urgent need for technological solutions to improve material quality specially for paper and plastic recyclables [26,27,28,29]. Simultaneously, the proliferation of single-stream recycling programs, while increasing participation rates, has introduced new challenges related to contamination management. These programs, which allow consumers to place all recyclable materials in a single container, have contributed to higher contamination rates due to consumer confusion and the mixing of materials during collection.

The convenience that makes these programs successful also creates technical challenges that require innovative solutions. Recent advances in computer vision and deep learning have created new possibilities for addressing complex detection tasks in industrial settings.

The remarkable success of CNNs in image classification and object detection across diverse applications [30,31,32,33,34,35,36,37,38,39] suggests untapped potential for applying these technologies to recycling challenges. Several pilot studies utilizing machine learning for waste sorting have demonstrated promising results, but comprehensive implementations for specific contamination issues remain limited. Additionally, growing awareness of plastic pollution and its environmental impacts has intensified interest in improving recycling effectiveness.

Technology that improves recycling outcomes thus contributes to addressing one of the most pressing environmental challenges of the current era. From an economic perspective, volatility in recycled material markets has created strong incentives for quality improvement. Facilities capable of producing consistently high-quality materials can command premium prices and maintain market access even during downturns. The financial motivation for contamination reduction has never been stronger, creating a receptive environment for technological innovation in this space.

### 1.5. Structure of the Study

The remainder of this paper is organized into five chapters that systematically address the research objectives and present the development, implementation, and evaluation of the CNN-based detection system for uncovered plastic bottles. Section 2 provides a comprehensive examination of relevant research across multiple domains, including recycling contamination challenges, existing detection and sorting technologies, applications of computer vision in waste management, and recent advances in CNN architectures for similar detection tasks. The review identifies knowledge gaps and establishes the theoretical foundation for the current research. Section 3 details the research design and technical approach employed in this study. This chapter describes the data collection procedures, dataset characteristics, CNN architecture, training protocols, and evaluation methodologies. Section 4 presents the empirical findings from model training and evaluation. This chapter reports on the detection accuracy, precision, recall, and processing speed under different operational scenarios. The analysis includes comparisons with baseline methods and examines performance. Section 5 discusses model deployment and its impact on the recycling industry. Section 6 summarizes the key research contributions, revisits the original objectives, and assesses the extent to which the developed system addresses the identified problem. This chapter also outlines directions for future research and development in this field.

## 2. Relevant Literature

Recycling systems worldwide face significant operational challenges, with contamination being one of the most critical issues affecting the efficiency and economic viability of recycling programs. Contamination in recycling streams refers to the presence of non-recyclable materials or recyclable materials that are compromised by residual contents, improper preparation, or cross-contamination with other materials.

SSR has emerged as the most popular and fastest-growing municipal recycling method in the United States due to its convenience for consumers and operational benefits for waste management companies. As Bafail [10] notes, SSR’s favored status rests on its minimal consumer burden, requiring only a cursory identification of potentially recyclable materials for placement in a single container separate from other waste. According to Moore [15], the number of SSR material recovery facilities (MRFs) increased in Florida from 5 facilities in 1995 to 287 in 2014, demonstrating the rapid growth of this approach. By 2014, 73% of the U.S. population with recycling programs had access to SSR programs, up from just 22% in 2005 [18,21].

Of particular concern for this study is the contamination caused by uncovered plastic bottles containing liquids, which can release their contents during collection and processing, creating cascading contamination issues that affect other recyclable materials.

Recent studies examining recycling contamination sources have highlighted the significant challenges posed by liquid residues in the waste stream [40]. These contaminants compromise material quality throughout the recycling process. Management of the large increase in plastic waste quantity has been particularly challenging, especially in regions experiencing rapid economic development and population growth where only 9% of plastic waste has been recycled globally, with the overwhelming majority of global plastic waste being landfilled or ending up contaminating the environment (80%) [40]. Resulting in an estimated 4 million to 12 million metric tons of waste plastic entering the oceans annually [41,42]. This contamination crisis underscores the urgent need for improved detection and sorting technologies.

Another study by Brouwer et al. [43] examining European PET bottle recycling systems reveals critical distinctions between mono-collection (PET bottles only) and co-collection approaches (mixed recyclables). Despite the increasing adoption of recycled content policies, plastic items frequently contaminate other materials during co-collection, particularly when residual liquids leach into paper and cardboard. This cross-contamination significantly complicates downstream sorting processes, reducing both recovery rates and material value. Scientific research on contaminant accumulation in PET recycling remains surprisingly limited, creating significant knowledge gaps in recycling optimization.

A previous study highlighted that the majority of post-consumer plastic waste remains unrecycled due to significant challenges in separation processes, contamination from impurities, and degradation of the macromolecular structures, all of which compromise the material integrity during conventional recycling approaches [44].

The accumulation of plastic waste, particularly from packaging and single-use items, has reached alarming levels, with only a small fraction being recycled globally [40]. The widespread use of plastics in various sectors, coupled with inadequate waste management practices, has led to significant environmental pollution [45]. Plastic waste poses a serious threat to ecosystems, wildlife, and human health [46]. The slow degradation rate of plastics results in long-term accumulation in landfills and natural environments, exacerbating the problem of plastic pollution [47]. It is, therefore, imperative to develop comprehensive strategies for reducing plastic waste generation, improving recycling rates, and promoting the use of sustainable alternatives.

### 2.1. Impact of Contamination in Recycling Economics and Operations

The economic implications of contamination in recycling streams are substantial and have been exacerbated by international policy changes. Until 2017, the United States shipped most of its recyclable materials to China for processing due to China’s flexible regulatory environment and low labor costs. However, in 2017, China announced its “National Sword Policy,” which dramatically restricted the importation of contaminated recyclable materials and required reduced contamination rates of 0.3% for acceptable materials [48,49,50,51,52,53,54,55].

These policy changes had immediate and severe economic impacts on the U.S. recycling industry. For example, mixed fiber recyclable material bale prices in the United States fell dramatically from average prices of USD 65–72 per ton in July 2017 to USD 17–37 per ton by September 2017, depending on the region [10]. By September 2019, the average prices of mixed-fiber recyclables had declined to zero in all regions of the United States. Similarly, old corrugated cardboard (OCC) prices fell significantly, from over USD 150 per ton in July 2017 to USD 15–37.5 per ton by May 2019 [10].

These market collapses directly reflect the international rejection of contaminated materials, illustrating how contamination not only destroys market value but fundamentally undermines recycling economics. High contamination of inbound streams results in prohibitive contamination rates in materials that are shipped to mills. In paper mills, if the prohibitive contamination rate exceeds the paper mill standard rates, the outbound paper materials become useless [13]. High contamination levels in the waste stream also increase the incidence of recycling equipment machine failure [56].

The economic sustainability of recycling operations depends significantly on contamination reduction and material recovery rates. The poor quality of the collected materials under different recycling stream approaches has rendered recycling no longer cost-effective for some municipalities [26,27,28].

This economic vulnerability largely stems from specific contamination vectors that disproportionately impact material quality, with liquid contamination representing one of the most financially destructive forms.

The specific issue of uncovered plastic bottles containing liquids represents a particularly problematic category of contamination. When these bottles are processed alongside paper, cardboard, and other recyclable materials, they release their liquid contents, resulting in widespread contamination that reduces the quality and value of recycled materials.

Yasar et al. [57] noted that mixing certain plastic materials such as HDPE, HDPE colored, PET, and film with other recyclables at the collection stage can cause contamination to the MRF inbound stream.

Rosbach [58] asserts that the U.S. currently recycles only 10% of its annual plastic waste. Rosbach identified a lack of proper education regarding recycling as the most significant factor contributing to low recycling rates in SSR. Raising participant awareness regarding separating recyclable and non-recyclable plastics is critical to reducing contamination rates.

### 2.2. Current Detection and Sorting Technologies in Recycling

The technological landscape of recycling sorting has evolved significantly over the past decade, yet substantial gaps remain in addressing specific contamination challenges. Conventional sorting technologies employ a variety of mechanisms, including mechanical, optical, and density-based approaches, each with distinct capabilities and limitations in detecting and removing contaminants.

**Figure 2:**
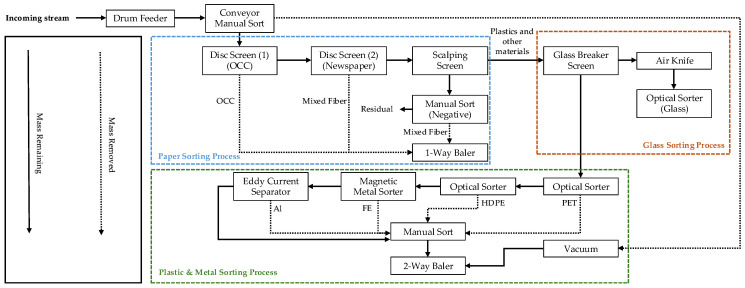
Material flow at MRFs [19].

Optical sorting represents the current industry standard for automated material identification in recycling facilities. These systems typically employ near-infrared (NIR) spectroscopy, visible light spectrum analysis, or a combination of these technologies to identify material types, as shown in Figure 2. While NIR sorting performance across various recycling facilities was found to be high [59,60,61,62,63,64,65], its effectiveness is substantially compromised by cross-contamination that occurs during the collection phase. When various materials are commingled in single-stream or mixed recycling systems, contamination has already taken place before materials reach sorting facilities. Liquid residues from uncapped containers, food waste, and other contaminants have already compromised material integrity, rendering even perfect identification less valuable since the separated materials remain contaminated. This fundamental limitation means that sophisticated sorting technology cannot fully compensate for contamination that occurs upstream in the recycling process.

X-ray fluorescence (XRF) technology plays a crucial role in modern plastic recycling facilities by enabling rapid, non-destructive identification of specific plastic types and contaminants. In MRFs, XRF sorters are primarily deployed to identify and remove PVC contaminants from PET streams, as well as to detect plastics containing brominated flame retardants (BFRs). The process is automated through conveyor systems where items pass through XRF sensors that trigger separation mechanisms when target materials are detected [66]. While XRF sorting is limited in distinguishing between all polymer types, it excels at identifying specific elements like chlorine (in PVC) and bromine (in flame retardants) [67,68,69], making it an essential component in ensuring the purity of recycled plastic streams.

Mechanical separation technologies, including trommels, disc screens, and ballistic separators, primarily target size and shape characteristics rather than material properties or container conditions. Researchers developed mechanical sorting techniques for plastics using centrifugal force, specific gravity, and the hybrid jig method combining jigging and flotation, improving separation efficiency beyond traditional methods [70]; however, these methods cannot reduce contamination already present at the collection stage. These technological gaps highlight the need for more sophisticated detection systems specifically designed to identify uncapped, liquid-containing bottles early in the sorting process before contamination occurs. Early applications of computer vision in waste management focused primarily on material classification. Researchers [71,72,73] implemented a vision-based system to distinguish between major waste categories (plastic, paper, metal, and glass) using conventional image processing techniques and achieved high classification accuracy under controlled conditions. However, those applications and models only separate materials at MRF rather than preventing contamination at the collection stage. The introduction of deep learning approaches, particularly CNNs, represented a significant advancement in waste classification capabilities. Bircanoglu et al. [74] implemented a CNN-based classification system for recyclable materials that achieved over 87% accuracy across multiple waste categories, substantially outperforming traditional machine learning approaches. Their implementation demonstrated improved robustness to variations in lighting, orientation, and partial occlusion. Building on this foundation, Kaya et al. [75] developed a more specialized CNN architecture specifically optimized for waste classification, achieving 89% accuracy in distinguishing between different recyclables under real-world conditions. Other studies achieved even higher accuracy in classifying and sorting recyclables at MRFs [76,77,78,79]. Recent research has expanded beyond simple classification to more complex object detection tasks, enabling the identification of specific items within mixed waste streams. Ziouzios et al. [80] implemented a faster R-CNN architecture to detect and localize diverse recyclable items on conveyor belts, achieving over 90%. Paz [81] extended this approach by implementing the YOLO (you only look once) object detection architecture specifically for colored glass bottles. The system achieved 88% detection accuracy. Thus, several scholars [82,83,84,85,86,86] have implemented YOLO object detection algorithms to revolutionize recyclable sorting in waste management facilities. These implementations have significantly improved both processing efficiency and classification accuracy. YOLO’s real-time processing capabilities allow for faster conveyor belt speeds while maintaining precise identification of various materials [87,88,89], including plastics, metals, and paper products. The technique’s ability to detect multiple objects simultaneously makes it particularly valuable in high-throughput recycling facilities, reducing labor costs and minimizing sorting errors. As computational resources become more affordable, YOLO-based sorting systems represent a promising technology for addressing the increasing demands of circular economic initiatives worldwide.

### 2.3. Research Gaps and Opportunities

The literature review reveals several significant research gaps and opportunities related to the CNN-based detection of uncapped plastic bottles in recycling streams. While existing models have demonstrated their effectiveness in general waste classification and sorting at material recovery facilities, there remains a critical absence of detection systems specifically designed to identify uncapped plastics at the earlier collection stage. This study addresses this gap by extending CNN implementations to detect uncovered plastic containers when waste is initially collected, enabling early intervention and contamination control. This approach represents a fundamental shift from reactive sorting to proactive contamination prevention, as identifying problematic items before they enter the processing stream can significantly improve recycling efficiency and end-product quality. By intervening at the collection point, potentially contaminated materials can be flagged for further inspection before compromising entire batches of recyclables.

## 3. Materials and Methods

**Figure 3:**
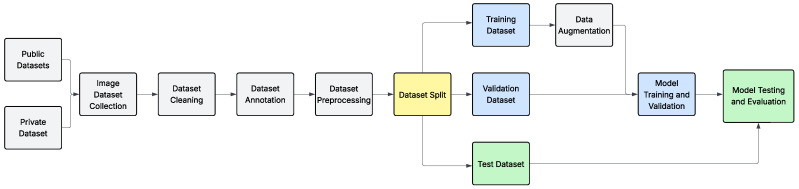
Research methodology workflow.

In this study, we utilized YOLOv8 (you only look once), a well-established one-stage object detection model. We trained the model using a standard deep learning method, as shown in Figure 3.

### 3.1. Dataset Creation and Preprocessing

**Figure 4:**
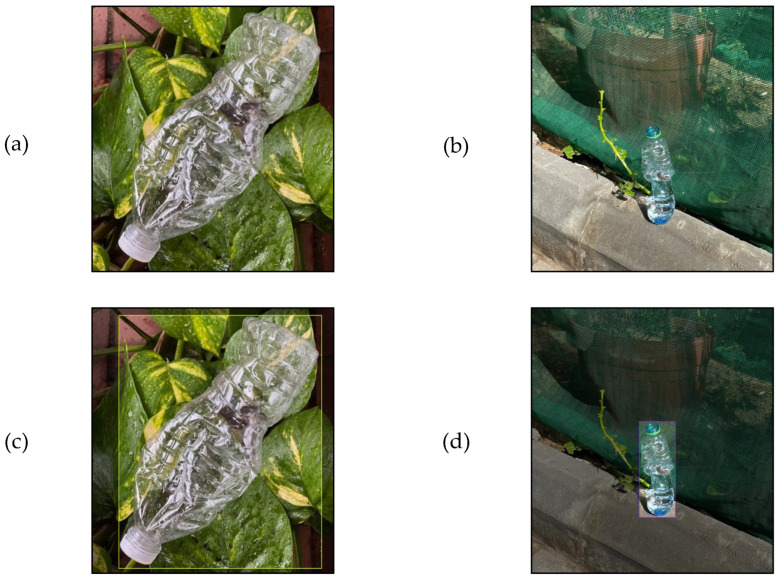
Sample of images in the dataset: (**a**) accepted class, (**b**) rejected class, (**c**) accepted class with annotation, and (**d**) rejected class with annotation.

Various transparent PET bottle images were collected using an iPhone 13Pro (Apple, Cupertino, CA, USA) with default settings. The image resolution was 3024 × 4032 pixels, and the images were taken under different natural lighting conditions, backgrounds, and orientations for various bottle sizes and liquid levels. To ensure the model’s robustness against different contaminant types, the dataset included PET bottles containing a range of liquids, such as clear, opaque, and colored. The total number of images collected for each class was 600, with at least one instance of a PET bottle in each image, as shown in Figure 4. Furthermore, two public datasets were used to increase the dataset size to 974 images [90,91]. Images chosen from the public datasets had no water marks, were non-synthetic, had transparent PET bottles, and the image size was equal to or larger than 640 × 640 pixels.

Each image was labeled manually using the Roboflow annotation tool [92], and the dimensional data were saved as text files. A total of 974 images (1330 instances) were annotated, with an average of 1.45 labels per image. Two classes were annotated with bounding boxes as follows (see Figure 4):

- Accept: Covered bottles with a cap or uncovered empty bottles.
- Reject: Uncovered PET bottles containing more than 50 mL of liquid.

For both classes, the annotation process involved drawing a bounding box around the entire PET bottle that met the specified criteria. Therefore, the model was trained to identify and localize the entire bottle deemed ’Accept’ or ’Reject’, rather than segmenting the liquid within the bottle.

Following the annotation process, auto-orient preprocessing was applied to the dataset, and images were resized to fit within 640 × 640 pixels to reduce training time [93]. The dataset was split into 70%, 15%, and 15% subsets, where the training data had 684 images, and the validation and test data had 145 images each, as shown in Table 1. Training images were used to train the YOLOv8 algorithm for PET bottle object detection. The validation subset was used to monitor and optimize the training progress. Test images were used to evaluate the model performance.

**Figure 5:**
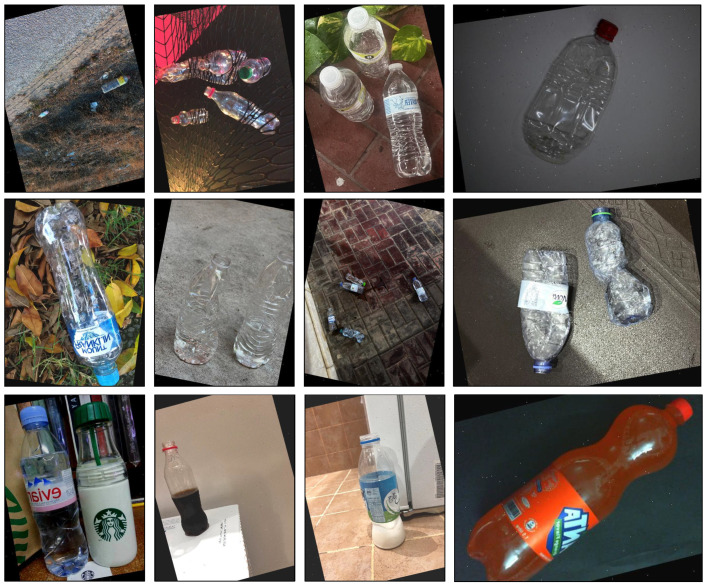
Sample of augmented images.

Moreover, training images were augmented 10-fold, increasing the set from 684 to 6840 images prior to training the model using the settings shown in Table 2. This brought the total number of images in the dataset to 7130 (see Table 1). Data augmentation is a standard technique in computer vision tasks to artificially enlarge the dataset, which helps the model learn more features and real-world scenarios and prevents it from overfitting the dataset [94,95,96,97,98]. To carefully examine the dataset quality, we chose to perform augmentation prior to training the model, and we disabled the augmentation on-the-fly option during training. A sample of augmented images from the dataset is shown in Figure 5.

### 3.2. YOLOv8 Object Detection Model

**Figure 6:**
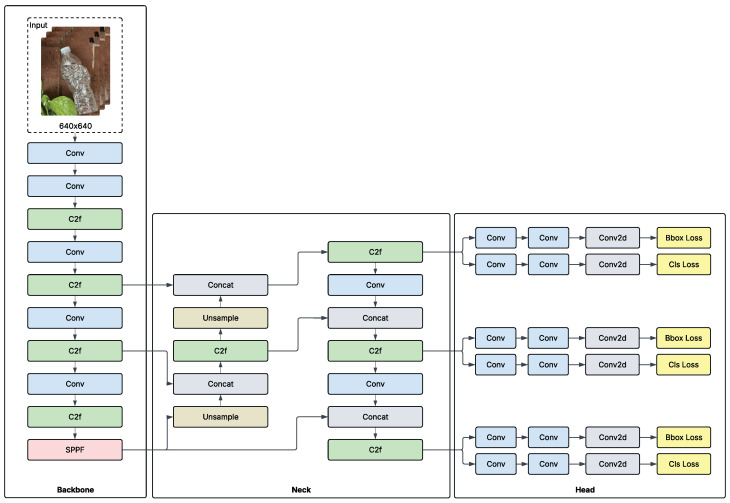
YOLOv8 original architecture [99].

In this study, we trained YOLOv8, which is a well-established real-time object detection model. It is a single-stage model with a convolutional neural network (CNN) backbone, as shown in Figure 6 [99]. The model has three main parts: the backbone, head, and neck. The backbone is used to learn features efficiently from input images. The neck of the architecture functions as an orchestrator by fusing information from different levels in the backbone. The head outputs the model’s final prediction by drawing bounding boxes and assigning probabilities to each class [100].

The architecture is one of several in the YOLO family of models, and it is available in different sizes: small, medium, large, and extra-large. In computer vision, there is typically a trade-off between model accuracy and model size or inference speed; as the model size increases, accuracy improves, but at the expense of model speed and size [100]. The size of the model is usually chosen based on the task requirements and model deployment device. In our study, we prioritized accuracy over model size and speed, as contamination caused by PET bottles can be costly for stakeholders.

### 3.3. Model Training

We trained YOLOv8l, where the “l” stands for the large size of the model, which has approximately 43 million parameters. We utilized a transfer learning approach for our training. This technique allowed us to employ a pre-trained version of the model and fine-tune it for our object detection task. The training went through 50 epochs with a patience of 30 and a batch size of 4 due to the limited capacity of the video random access memory (VRAM). Bulit-in augmentation was disabled during training as we manually augmented the data prior to training. The model was optimized using the AdamW algorithm with a learning rate of 0.00167, a momentum of 0.9, and a weight decay of 0.0005.

The hyperparameters for training the models, summarized in Table 3, were selected to establish a robust baseline and ensure stable convergence on our custom dataset. The AdamW optimizer was utilized, a choice automatically determined by the Ultralytics framework’s default settings, which are favored for their adaptive learning rate capabilities and robust performance in deep learning tasks. The associated learning rate of 0.00167, momentum of 0.9, and weight decay of 0.0005 were the default values for this optimizer, providing a proven and effective starting point for fine-tuning without extensive manual tuning. The Image size was set to 640 × 640 pixels, a standard resolution for many state-of-the-art object detection models, including YOLO, as it offers a strong balance between retaining sufficient feature detail for accurate detection and managing computational load. The batch size was set to four, a practical necessity determined by the hardware constraints. The training was conducted for a maximum of 50 epochs to allow the model sufficient passes over the data to converge. Also, to prevent overfitting and save computational resources, an early stopping mechanism was implemented with a patience of 30 epochs. This value was chosen to provide a generous window for the model to overcome potential performance plateaus and find its true peak on the validation set before halting the training process.

Training was performed on Google Colab Pro Plus using an NVIDIA A100 GPU with 40 GB of VRAM, PyTorch 2.6, and CUDA 12.4. Furthermore, we employed the Ultralytics YOLO framework version 8.3.137 [99].

### 3.4. Evaluation Metrics

Standard evaluation metrics, such as precision (P), mean average precision (mAP), recall (R), and intersection over union (IoU), were used to assess the model performance in accepting or rejecting PET bottles [101,102]. These standard object detection metrics were calculated using Equations (1) to (5).

The precision metric measures the model’s ability to make correct predictions out of the total number of positive detections. It is an indicator of model accuracy and can be calculated using Equation (1), which divides the true positive (TP) values by the sum of TPs and false positives (FP).

(1) $$\mathrm{Precision}=\frac{\mathrm{True}\;\mathrm{Positive}\;\left(\mathrm{TP}\right)}{\mathrm{True}\;\mathrm{Positive}\;\left(\mathrm{TP}\right)+\mathrm{False}\;\mathrm{Positives}\;\left(\mathrm{FP}\right)}.$$

The mAP measures the average precision (AP) value across all N classes. This metric is the most commonly reported, as it provides a comprehensive indicator of model performance and can be calculated using Equation (2):

(2) $$\mathrm{mAP}=\frac{1}{N}\sum_{i=1}^{N}\left({\mathrm{AP}}_{i}\right).$$

The recall (R) metric, also known as sensitivity or true positive rate, measures the proportion of actual positive instances that were correctly identified by the model. It is calculated by dividing TPs by the total of the TP and false negative (FN) values, as shown in Equation (3):

(3) $$\mathrm{Recall}=\frac{\mathrm{True}\;\mathrm{Positive}\;\left(\mathrm{TP}\right)}{\mathrm{True}\;\mathrm{Positive}\;\left(\mathrm{TP}\right)+\mathrm{False}\;\mathrm{Negatives}\;\left(\mathrm{FN}\right)}.$$

The F1 score metric provides a balanced or harmonic mean between precision and recall values. A high F1 score generally indicates effective performance in the detection and classification of bottles. The metric can be calculated using Equation (4):

(4) $$F1=\frac{2\times \mathrm{Recall}\times \mathrm{Precision}}{\mathrm{Precision}+\mathrm{Recall}}.$$

The intersection over union is an essential measure in object detection, as it measures the overlap of the predicted bounding box with the ground truth bounding box. A higher value indicates a greater degree of overlap, which, in turn, suggests a more accurate localization prediction. In our study, we report a mAP with IoU of 0.5 (mAP@0.5) and IoU from 0.5 to 0.95 (mAP@0.5:0.95). The IoU can be calculated using Equation (5):

(5) $$\mathrm{IoU}=\frac{\mathrm{Area}\;\mathrm{of}\;\mathrm{Overlap}}{\mathrm{Area}\;\mathrm{of}\;\mathrm{Union}}.$$

Inference speed is another essential metric for evaluating object detection models for real-time capabilities. It is typically influenced by the model size and architectural design and is measured in ms/image.

## 4. Analysis and Results

**Figure 7:**
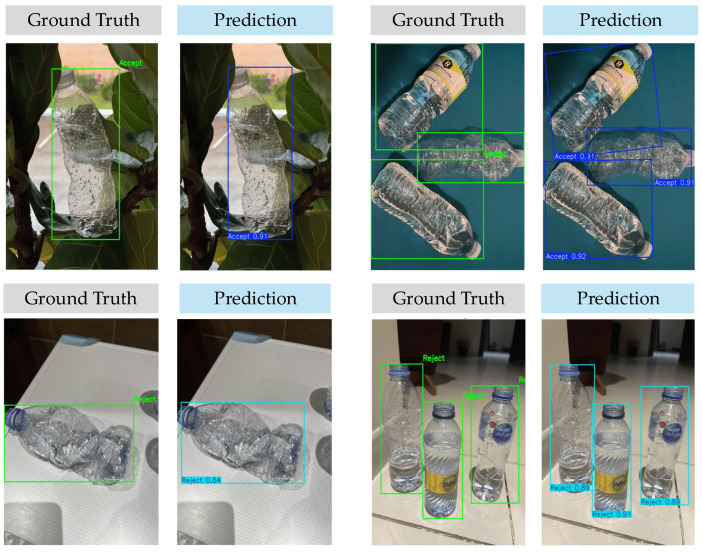
PET bottle object detection results on test images.

The model evaluation results for the training and test sets are presented in Table 4. According to the findings, the model achieved 89% mAP@0.5:0.95 in detecting PET bottles. Precision in both classes reached 91%, which indicates that most of the model predictions were correct. While recall for both classes was 85%, indicating that the model missed some rejected bottles. Given the well-balanced distribution of instances between the ‘Accept’ and ‘Reject’ classes in the training dataset shown in Table 4, specialized loss-weighting or data resampling techniques to address class imbalance were not required for this phase of the study. Overall, the model was slightly better at detecting acceptable bottles, which could be due to class imbalance in the test set. However, 92% mAP@0.5, as shown in Figure 7, is an excellent object detection result.

**Figure 8:**
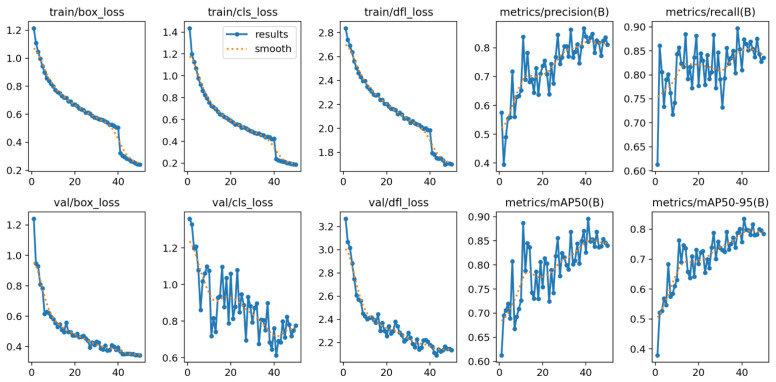
YOLOv8l training results and evaluation metrics (the horizontal axis for all 10 subplots represents epochs, the vertical axis represents the metric value, and the labels for each Y-axis are indicated by the title of each subplot).

**Figure 9:**
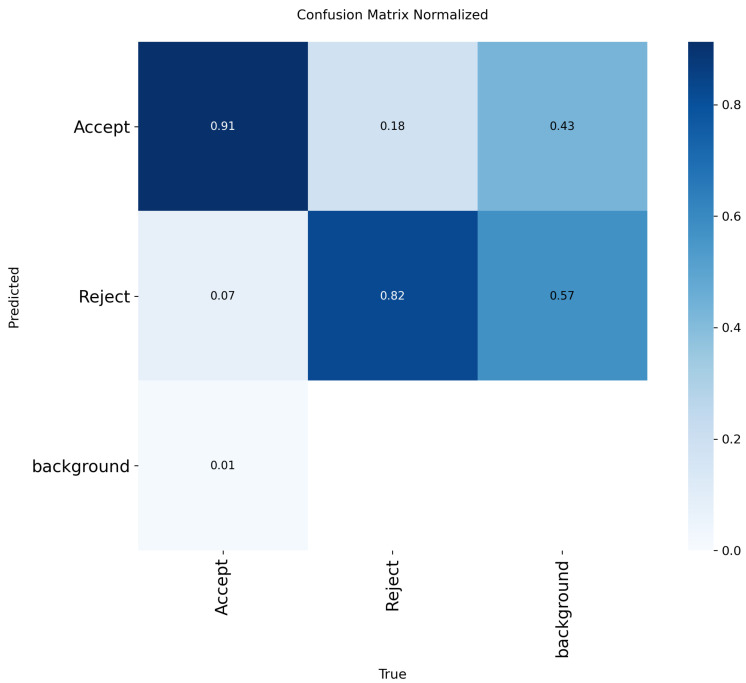
Confusion matrix for YOLOv8l.

In Figure 8, we show the model results for training, validation loss, and metrics evaluation during 50 epochs. The model converged within 50 epochs, and generally, all the loss curves decreased and then plateaued. Also, all performance metric curves increased and then plateaued at a high value, which indicates successful and stable training. The confusion matrix shown in Figure 9 highlights the model’s ability to detect true positive values (0.91–082) and effectively differentiate between accepted and rejected bottles. These results further support the idea that YOLO models are capable of detecting PET bottles [103,104,105].

YOLOv8n was designed to prioritize speed over accuracy, where “n” stands for the nano version of the model. On the other hand, YOLOv8x is designed with the highest accuracy in mind. YOLOv8l strikes a good balance for our specific application as preventing contamination by detecting rejected bottles correctly is essential. Table 5 shows the performance of different model sizes on the test set.

Five-fold cross-validation was conducted to reduce the risk of overfitting and strengthen the model generalization [106]. Cross-validation was carried out by splitting the dataset into several subsets, with one set held out as the test set. During the five folds or iterations of the cross-validation, a different test set was chosen for each fold, and then the average of all folds ws calculated, as shown in Table 6. The mAP@0.5:0.95 after cross-validation was 89.7%, which is similar to the score achieved from a single split evaluation. YOLOv8l achieved mAP50, precision, recall, and F1 score values of 0.93, 0.89, 0.88, and 0.88, respectively, on a custom PET bottles dataset. These results confirm the model’s generalization and ability to detect different bottles under various conditions.

YOLOv8l achieved the best balance of accuracy, inference speed, and model size, as shown in Table 7. It significantly improves upon YOLOv5l in all metrics except recall, and it outperforms newer variants from the YOLO family, such as YOLOv11l and YOLOv12l, suggesting that it remains the optimal choice among the tested models for real-time, high-performance applications. Future studies could explore increasing the dataset size and ensembling YOLO models to further improve performance.

## 5. Discussion

The Discussion section examines implementation strategies for the proposed YOLOv8l model in addressing the critical challenge of liquid contamination in recycling streams. This contamination represents a multifaceted problem for MRFs worldwide, as uncovered liquid-containing bottles compromise material integrity, damage equipment, and significantly reduce the market value of recyclables. The economic ramifications are particularly severe, as evidenced by the precipitous decline in old corrugated cardboard prices following China’s implementation of stringent quality standards, a 75–90% value reduction that fundamentally threatens recycling program viability. Beyond financial considerations, the environmental implications extend to increased landfill usage, methane emissions, and unnecessary virgin resource consumption when contaminated materials cannot be processed effectively.

**Centralized Deployment**: The primary application context for this system is at the initial intake stage of an MRF. By deploying the system over the main conveyor belt carrying the mixed recycling stream, contaminated PET bottles can be identified and removed before they enter downstream processes. High-speed industrial cameras would be mounted above the conveyor, housed within an enclosure featuring consistent, high-intensity LED lighting. This controlled environment mitigates issues arising from variable ambient lighting commonly found in MRFs. An industrial computer with a suitable GPU would process the camera feed in real time, running the YOLOv8l model to output the class and bounding box coordinates for each detection at a frame rate sufficient for the conveyor’s speed. The software application would intake the video stream, perform inference, and output the class and bounding box coordinates for each detected bottle. The output of the model will guide the physical removal of targeted contaminated objects. Thus, two physical integration strategies are proposed: (1) Robotic Sorting System: For higher precision, the system can be integrated with a robotic arm. In this configuration, the model provides the precise coordinates and orientation of the target bottle to a robot control system. This approach offers great efficiency and can be adapted to handle a wider variety of object shapes and weights. (2) Human-in-the-loop (HITL) System: A practical and lower-cost alternative, the system can function as a powerful decision-support tool for manual sorters. This HITL approach leverages the tireless consistency of AI detection to augment the judgment of human workers, significantly increasing their efficiency and accuracy without the capital investment of a fully robotic system.

The implementation of this system yields multifaceted advantages across operational, economic, and environmental dimensions. The upstream interception of liquid contaminants fundamentally transforms MRF processing dynamics by preserving the integrity of fiber-based materials, which constitute approximately 67% of the recycling stream by volume. Rather than the conventional reactionary approach to contamination, which necessitates downstream separation and remediation, this pre-emptive strategy significantly reduces moisture-related degradation of paper and cardboard commodities. Quantitatively, facilities implementing similar early detection technologies have documented reductions in moisture content from 24–28% to below 12% in output bales, substantially elevating market valuations. The concomitant decrease in secondary cleaning operations could translate to approximately 0.34–0.47 kWh energy savings per ton processed, yielding cumulative reductions in both operational expenditures and carbon footprint.

Moreover, this technology fosters an evolutionary shift in recycling industry paradigms through its inherent adaptability. The foundational YOLOv8l architecture permits continuous refinement through transfer learning protocols, enabling the system to progressively expand its detection capabilities beyond liquid-containing PET bottles to encompass additional contaminant categories. This scalability circumvents the conventional requirement for wholesale equipment replacement when addressing emerging contamination challenges. For MRF operators navigating the increasingly stringent quality thresholds imposed by downstream processors, this represents a crucial competitive advantage. The integration also catalyzes workforce transformation, elevating sorter roles from repetitive manual tasks to supervisory positions that leverage human contextual intelligence while mitigating occupational health concerns associated with prolonged exposure to contaminated materials.

**Decentralized Deployment**: The implementation of a decentralized approach represents a paradigm shift in contamination management strategy by embedding the YOLOv8l detection model directly into intelligent recycling receptacles positioned throughout high-traffic public environments. Strategic placement in transportation hubs, athletic venues, commercial centers, educational institutions, and municipal parks creates an extensive network of prevention-focused nodes operating at contamination’s origin point. Each smart receptacle incorporates three essential components: a miniaturized optical sensor positioned at the deposit aperture, an energy-efficient edge computing module capable of executing the inference algorithm locally, and an interactive digital interface for user engagement.

The proposed operational workflow would embody intuitive human–technology interaction principles designed to maximize user engagement and learning. As recyclable materials would approach the deposit aperture, the optical system could capture visual data for immediate analysis by the edge processor. Upon detection of liquid-containing bottles, we suggest the system should initiate user notification through the digital interface, providing clear guidance on proper preparation before recycling. This real-time intervention would occur within the critical decision window when users could still modify their behavior, potentially creating an educational moment precisely when it holds maximum relevance. Additionally, the system could simultaneously record anonymized data regarding contamination patterns, building comprehensive analytics that might inform future prevention strategies and educational campaigns. This suggested interaction framework represents one potential implementation pathway that balances technological capabilities with behavioral psychology principles to address contamination at its source.

This approach yields substantial advantages across multiple domains. From an educational perspective, the immediate contextual feedback transforms abstract recycling guidelines into concrete, actionable information precisely when users require guidance. This point-of-decision intervention represents a significant advancement over traditional passive educational campaigns, which frequently fail to influence behavior at critical moments. The system effectively functions as an omnipresent recycling educator, delivering consistent instruction across diverse public environments without requiring additional human resources.

From operational and economic standpoints, addressing contamination at its origin fundamentally alters downstream processing dynamics. By preventing liquid contaminants from entering the recycling stream initially, the system preserves material integrity throughout the entire recovery chain. This pre-emptive intervention substantially reduces moisture-related degradation of paper and cardboard materials, maintaining their market value and reducing processing complexity at recovery facilities. Material recovery facilities receiving input from areas with deployed smart bin networks experience measurably reduced contamination rates, lower operational costs, and enhanced commodity values, creating economic incentives that support system adoption.

The environmental benefits extend beyond improved material recovery. By optimizing recycling streams, the system reduces landfill diversion of otherwise recoverable materials compromised by contamination. The consequent decrease in secondary cleaning operations conserves energy throughout processing cycles, reducing both operational expenses and environmental impact across the recovery continuum. Additionally, the data collected from distributed bins provides unprecedented visibility into contamination patterns, enabling targeted community education and system refinements that continuously improve performance over time.

Perhaps most significantly, this decentralized approach creates a participatory recycling ecosystem that transforms users from passive contributors into engaged stakeholders. The immediate feedback loop cultivates environmental literacy through practical application rather than abstract instruction. This experiential learning builds lasting behavioral changes that extend beyond individual interactions with smart receptacles, gradually establishing contamination prevention as a normalized social practice. The cumulative effect represents a foundational shift toward a more informed, environmentally conscious recycling culture that strengthens the entire materials recovery system.

## 6. Conclusions

This research offers a promising advancement in tackling the pervasive issue of liquid contamination within recycling streams, a constant operational hurdle for material recovery facilities (MRFs). The study centered on engineering a highly effective object detection system specifically designed to identify problematic PET bottles, which are frequent culprits in this type of spoilage. To achieve this, a range of YOLO model architectures and sizes were rigorously trained using a purpose-built dataset comprising 7130 images. Among the models tested, the YOLOv8l variant emerged as particularly proficient. It demonstrated a strong capacity for recognizing unwanted items, with a specific knack for detecting uncapped PET bottles that still contained liquids, marking these for “Reject” status. This system’s capabilities are underscored by its impressive performance figures: it achieved a mean average precision (mAP@0.5:0.95) of 89.7%, a mAP50 of 93%, coupled with a precision of 89%, a recall of 88%, and an F1 score of 88%. The proven, real-time accuracy of this YOLOv8l model makes it a highly practical tool for deployment in the initial phases of the recycling pathway, such as during material collection or as items enter the sorting process. Consequently, this work represents a tangible step forward in diminishing the impact of liquid contaminants in single-stream recycling environments, directly addressing a pressing need within the modern recycling industry.

The promising results achieved in this study lay the groundwork for several key research and development directions aimed at enhancing the system’s capabilities and moving it closer to industrial deployment. The primary next step is to transition the developed model into a centralized system. This involves integrating the real-time detection output of the YOLOv8l model with a physical system on a conveyor belt. Future work will focus on developing and testing this “detect-and-eject” pipeline with physical sorting mechanisms, such as robotic arms or HITL, to validate the system’s performance in a simulated operational environment.

Moreover, exploring semantic segmentation models represents a valuable avenue for improving the granularity of detection. While the current approach effectively identifies the entire PET bottle as contaminated, a segmentation model could precisely delineate the liquid portion itself. This would enable more sophisticated classification and provide a more robust trigger for the sorting mechanism.

A significant extension of this research could involve adapting the detection framework to address additional contamination vectors beyond liquid-containing bottles. Fragmented glass represents a particularly pernicious contaminant within recycling streams, as glass shards embed themselves in fiber-based materials and compromise downstream processing equipment. Future iterations could incorporate specialized detection parameters calibrated to identify the distinctive visual signatures of broken glass amid commingled recyclables, potentially leveraging light refraction patterns and edge detection algorithms to distinguish glass fragments from other materials. This expansion would address multiple contamination pathways simultaneously, substantially enhancing the system’s utility across diverse MRF environments.

## Figures and Tables

**Table 1:** Dataset splits with and without augmentation.

Dataset Split	Without Augmentation	With Augmentation
Training	684	6840
Validation	145	145
Test	145	145
Total	974	7130

**Table 2:** Data augmentation settings.

Augmentation Type	Settings
Flip	Horizontal, Vertical
Rotation	15° and +15°
Hue	−10° and +10°
Saturation	−25% and +25%
Brightness	−15% and +15%
Exposure	−10% and +10°
Blur	Up to 2.5 px
Noise	Up to 0.1% of pixels

**Table 3:** YOLOv8 hyperparameters for object detection.

Hyperparameter	Default Value
Image Size	640
Learning rate	0.00167
Batch size	4
Epochs	50
Momentum	0.9
Weight decay	0.0005
Optimizer	AdamW
Patience	30

**Table 4:** Performance evaluation of YOLOv8l for detecting PET bottles.

Class	Instances		Precision		Recall		mAP0.5		mAP0.5–0.95	
	Train	Test	Train	Test	Train	Test	Train	Test	Train	Test
**All**	9339	205	0.9992	0.9109	0.9982	0.8515	0.995	0.9216	0.978	0.8905
**Accept**	4900	149	0.998	0.957	0.999	0.907	0.995	0.956	0.974	0.91
**Reject**	4439	56	0.99	0.864	0.997	0.796	0.995	0.888	0.982	0.871

**Table 5:** Comparison of YOLOv8 model results.

Model	mAP@0.5:0.95	mAP@0.5	Precision	Recall	F1 Score	Inference Speed (ms/Img)
**YOLOv8n**	0.7174	0.767	0.6685	0.8425	0.7336	2.27
**YOLOv8s**	0.7053	0.7658	0.7456	0.76	0.7465	3.73
**YOLOv8m**	0.8249	0.8644	0.7887	0.8682	0.8263	4.70
**YOLOv8l**	0.8905	0.9216	0.9109	0.8515	0.8801	3.98
**YOLOv8x**	0.8654	0.9157	0.8775	0.8397	0.8566	6.57

**Table 6:** K-fold cross-validation results.

Fold	mAP@0.5:0.95	mAP@0.5	Precision	Recall	F1 Score
**Fold 1**	0.8963	0.9297	0.8992	0.8861	0.8925
**Fold 2**	0.8992	0.9429	0.8809	0.9011	0.8908
**Fold 3**	0.8922	0.9278	0.9004	0.8889	0.8946
**Fold 4**	0.8927	0.916	0.8816	0.8693	0.8753
**Fold 5**	0.9092	0.9423	0.9312	0.8547	0.891
**Average**	**0.897 ± 0.006**	**0.931 ± 0.01**	**0.898 ± 0.0183**	**0.880 ± 0.016**	**0.888 ± 0.006**

**Table 7:** Comparison of YOLO model results (the best results are in bold).

Model	mAP@0.5:0.95	mAP@0.5	Precision	Recall	F1 Score	Inference Speed (ms/Img)
**YOLOv5l**	0.7993	0.8924	0.8047	**0.8869**	0.8438	4.2
**YOLOv8l**	**0.8979**	**0.9317**	**0.8987**	0.8800	**0.8888**	**3.98**
**YOLOv11l**	0.8552	0.8993	0.8975	0.8528	0.8746	7.9
**YOLOv12l**	0.8388	0.9014	0.8304	0.849	0.8396	7.9

## Data Availability

The raw data supporting the conclusions of this article will be made available by the authors on request.

## Abbreviations

The following abbreviations are used in this manuscript:

AI	Artificial Intelligence
AP	Average Precision
BFRs	Brominated Flame Retardants
CNNs	Convolutional Neural Networks
FN	False Negatives
FP	False Positives
IoU	Intersection over Union
mAP	Mean Average Precision
MCS	Commingled Collection Systems
MRFs	Material Recovery Facilities
MWR	Mixed Waste Recycling
OCC	Old Corrugated Cardboard
P	Precision
PET	Polyethylene Terephthalate
R	Recall
SSR	Single-Stream Recycling
TP	True Positives
VRAM	Video Random Access Memory
YOLO	You Only Look Once
